# Accidents exposing blood to the staff of a hospital and university establishment in Algeria: Assessment and risk factors

**DOI:** 10.4314/ahs.v22i4.69

**Published:** 2022-12

**Authors:** Dalia Kheira Derkaoui, Abdessamad Dali-Ali, Zouleykha Abdelaziz, Nori Midoun, Mohamed Zina

**Affiliations:** 1 University Oran 1 Ahmed Ben Bella, Faculty of Science; 2 University Oran 1 Ahmed Ben Bella, Faculty of Medicine; 3 Etablissement Public des Soins de Proximité Boutelellis

**Keywords:** Accidents exposing blood, hospital staff, Algeria

## Abstract

**Background:**

Accidents exposing to blood AEB represent real public health problem in healthcare establishments. The objective of our study was to estimate the frequency of AEB As at our establishment as well as the risk factors that determine their occurrence.

**Patients and Methods:**

A cross-sectional descriptive survey was conducted at a hospital university establishment over period from October 16 to December 3, 2018. The survey concerned accident exposing blood to the staff of our establishment. Data entry and analysis was carried out using Epi-Info software.

**Results:**

A clear predominance of women was noted (79.2%) among the study population with a Sex ratio equal to 0.26. The average age was 27.7 ± 6.2 years.

The frequency of exposure to AEB among hospital staff was 48.5%. Needlestick injuries were the most common accident (88.3%), followed by splashing blood or body fluids (51.7%), and cutting with a sharp object (10.0%).

Among the risk factors significantly associated with the occurrence of AEB, we can cite the medical profession (OR = 3.94; p <0.001), the surgical specialty (OR = 3.3; p <0.01), the male sex (OR = 3.7; p <0.01). Likewise, risk of AEB increased significantly with age (p <0.01) and professional seniority (p <0.02)

## Introduction

Accidents exposed to blood (BEA) represent a real public health problem, especially among health professionals^1^. These accidents refer to any contact with blood or any other biological fluid containing blood, occurring on the occasion of a break in the skin (puncture or cut) or projection on the mucous membrane (eye, mouth, nose) or on damaged skin 2. The management of BEA and the resulting complications is a heavy burden in terms of costs, absenteeism and psychological impact.

Note that the risk of seroconversion after a needle stick is 0.3% for HIV, 3% for hepatitis C, and 30% for hepatitis B 1, 3, 4.

Indeed, in developing countries, 40 to 65% of viral hepatitis B and C, recorded among health personnel, would be attributable to percutaneous accidents. This proportion would be much lower in developed countries where the attributable fraction is less than 10% for viral hepatitis B^5^. In Algeria, few studies on BEA have been published apart from the work of Baghdadli which showed that BEA by needle stick was the most common type of accident in hospitals 6–8.

Thus, the objective of our work was to estimate the frequency of BEA in our establishment as well as the risk factors that determine their occurrence.

## Material and methods

A descriptive cross-sectional survey was carried out at the level of the hospital and university establishment of Oran, over a period ranging from October 16 to December 03, 2018.

The study had two parts, one of which focused on BEAs. All specialties as well as all professional categories were included in the study, with the exception of personnel having no contact with patients, such as medical secretaries or administrative officers.

Data entry and analysis were carried out using Epi-Info software with prior coding of the various responses. The descriptive analysis consisted in presenting the results of the qualitative variables in the form of percentages and of the quantitative variables in the form of averages. The study of the relationship between two qualitative variables was made using the Chi-square test of homogeneity. Similarly, the Student test was used for the comparison of two means.

The determination of the BEA risk factors was made on the basis of the calculation of the Odds Ratio (OR) with their respective confidence intervals. A relationship was considered significant for a threshold of p ≤ 0.05.

## Results

In total, a sample of 130 medical and paramedical personnel was surveyed. The average age of the study population was 27.7 ± 6.2 years with a significant difference between the two sexes (male sex = 30.4 ± 7.6 years versus female sex = 26.9 ± 5.5 years; p < 0.05).

A clear female predominance was observed (79.2%) with a sex ratio of 0.26.

The category of doctors represented 46.9% of all staff questioned, followed by nurses and nursing assistants with respective percentages of 27.7% and 14.6%.

Similarly, medical specialties came first (49.2%), followed by obstetrics gynecology (29.2%), and surgical specialties (10.8%).

During their professional career, 48.5% of hospital staff was exposed, at least once, to an AES. The most common type of accident was a needle stick (40.8%), followed by blood or body fluid splash (23.8%) and a cut by a sharp object (4.6%) (Table I).

**Table I:** Study population and accident exposure to blood

**Professional categories**	**Nbre**	**%**
Doctors	61	46.9
Nurses	36	27.7
Caregivers	19	14.6
Midwives	11	8 ,5
Others	3	2.3
**Specialties**	**Nbre**	**%**
Medical	64	49.2
Obstetric gynecology	38	29.2
Chirurgicale	14	10.8
Intensive care	6	4.6
UMC	3	2.3
Is not pronounced	5	3.9
**Training on standard precautions**	**Nbre**	**%**
Oui	73	56.1
Non	46	35.4
Is not pronounced	11	8.5
**Date of last training**	**Nbre**	**%**
≥ 5 ans	48	65.8
> 5 Non	18	24.7
Is not pronounced	7	9.6
**Exposure to an AES**	**Nbre**	**%**
Yes	63	48.5
No	62	47.7
Is not pronounced	5	3.8
**Types of AES**	**Nbre**	% *
Sting	53	40.8
Projection	31	23.8
Cut	06	4.6

* % obtained by relating the number

f modalities to 130

In monofactorial analysis, the factors significantly associated with the risk of AES were medical profession (OR = 3.94), male sex (OR = 3.65), age over 27 years (OR = 3.33), surgical specialty (OR = 3.28), and seniority greater than 2 years (OR = 3.4) (table II).

**Table II:** Risk factors related to BEA

		Accident exposing to blood				

Risk factors		Yes Nbre (%)	No Nbre (%)	OR	IC a 95%	p
**Occupation**	**Medical**	40 (63.5)	19 (30.6)	**3.94**	**(1.87 – 8.29)**	**< 0.001**
	**Paramedical**	23 (36.5)	43 (69.4)			

**Sex**	**Male**	20 (31.7)	7 (11.3)	**3.65**	**(1.41 – 9.44)**	**< 0.01**
	Feminine	43 (68.3)	55 (88.7)			

**Age**	**> 27 years**	34 (59.7)	16 (30.8)	**3.33**	**(1.51 – 7.34)**	**< 0.01**
	**– 27 years**	23 (40.3)	36 (69.2)			

**Specialty**	**Chirurgical**	36 (59.0)	18 (30.5)	**3.28**	**(1.54 – 6.97)**	**< 0.001**
	**Medical**	25 (41.0)	41 (69.5)			

**Seniority**	**>2 years**	32 (66.7)	10 (37.0)	**3.4**	**(1.27 – 9.10)**	**< 0.05**
	**≤2 years**	16 (33.3)	17 (63.0)			

**Training** 1	**No**	36 (51.4)	18 (40.0)	**1.6**	**(0.74 – 3.39)**	**0.23**
	**Yes**	34 (48.6)	27 (60.0)			

1 Training in standard precautions

In terms of knowledge assessment, the indication for washing the skin wound with soap and water, in the event of an injury, was only known by 35.4% of the staff, unlike Dakin disinfection cited by 75.4 % of our sample.

**Figure 2:**
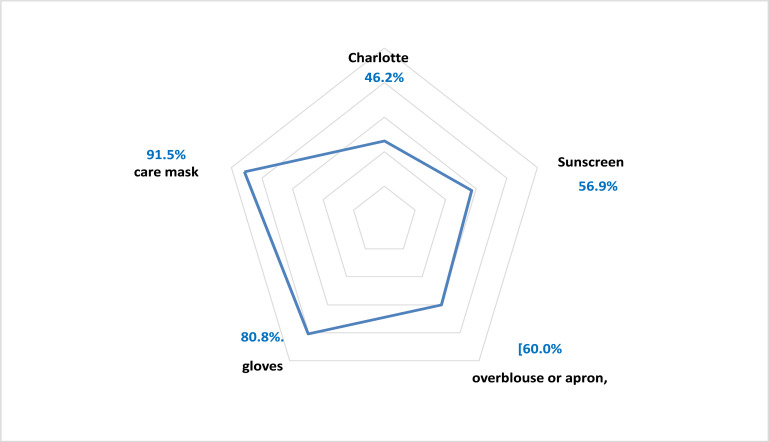
Knowledge of personal protective equipment in case of risk of splashing blood or any other biological fluid

In the event of a risk of projection, the treatment mask was the most cited personal protective equipment (91.5%), while protective goggles were only known by 56.9% of the staff (figure 2).

In terms of the management of soiled equipment, 57.5% of hospital staff think that sharp, cutting or edging equipment should be discarded immediately, without recapping, in a suitable and puncture-resistant container. In addition, 70.3% declare that the transport of biological samples, soiled linen and soiled materials must be done in a sealed and closed packaging.

## Discussion Frequency

In our study, exposure to AES was reported by 48.5% of the hospital staff questioned. This proportion was lower than that found in the Moroccan study by Laraqui, in which 76.6% of the staff had been exposed to an AES, at least once, during their career 9.

However, it should be noted that the frequency of AES varies from one region to another. In Bosnia, 54.8% of healthcare personnel had been exposed to ESAs in 2013. The average exposure during all years of practice was 7 accidents for each healthcare personnel 10. Similarly, the frequency of exposure to AES was 17.5% among medical students in Casablanca 11.

### Risk factors

The different professional categories concerned by AES differ according to the studies. In ours, doctors were almost 4 times more exposed than paramedics (p < 0.001), whereas in Jahic's study, nurses were significantly more affected 10.

Similarly, BEAs in our study were significantly more frequent in surgical specialties (OR = 3.3) unlike Bouhlel's study in which BEAs occurred preferentially in medical departments 12.

Also, seniority greater than 2 years was significantly associated with the risk of BEA in our study with an OR of 3.4. Indeed, according to some studies, health personnel feel more confident with the acquisition of a certain professional experience, and consequently, they become less vigilant in the face of risky gestures 13. For other studies, the risk of BEA decreases proportionally with seniority in the position due to the professional experience acquired over time 14.

### Types of Injuries

At our establishment, needle sticks were the most common accident (40.8%), followed by blood or body fluids splashing (23.8%).

Worldwide, percutaneous accidents vary on average from 0.2 to 4.7 percutaneous injuries per healthcare worker per year, with disparities depending on the region 5. Several studies cite needlestick as the most common cause 7, 15. These accidents are mainly linked to certain risky procedures such as recapping needles 9, 11. It should be noted that compressing the injured finger should not be done because it promotes the dissemination of the inoculum by transforming a superficial wound into a deep wound 16. In our study, 27.7% of the personnel questioned thought that this gesture was part of the conduct to be adopted in front of a BES.

Furthermore, the risk of splashing blood or biological fluids should not be neglected, as shown by the studies of Bambenongama and Nouetchognou in which these accidents represented 61.1% and 60.3% of cases respectively 1, 16.

### Germ Risk

After BEA, all microbes can be transmitted from the source patient to healthcare personnel (virus, bacteria, fungus or parasite) 17. According to the WHO, BEAs are responsible for 2.5% of HIV cases and 40% of viral hepatitis B and C cases among healthcare workers 18.

The risk of contamination is mainly linked to the prevalence of the disease among patients and the chronic nature of the latter 19. The risk also depends on the number of microbes inoculated which increases proportionally with the diameter and the depth of the wound, especially if the latter is caused by a hollow needle or one containing fresh blood 2, 19.

### Role of the HBV vaccine

Before initiating vaccination, the prevalence of hepatitis B was 2 to 10 times higher among health workers than in the general population 20, 21. In rich countries, good vaccination coverage has significantly reduced the risk of the disease among healthcare workers, like the United States of America, which has seen the incidence of hepatitis B drop by 95%, from 1983 to 1995 22–24. After anti-HBV vaccination, personnel are only considered protected if the titration of anti-HBs antibodies is greater than 100 IU/l 25.

For viral hepatitis C and HIV, no vaccine is currently available, hence the importance of insisting on standard precautions. These were updated by the French hospital hygiene society in 2017 26. The purpose of these precautions is to minimize any risk of contact with blood or other body fluids. In our study, the indications for wearing a mask and protective glasses, in the event of a risk of projection, were cited by 91.5% and 56.9% of the personnel questioned respectively.

We should also underline the interest in of the good management of the DASRI as well as the use of invasive devices provided with a protection system system allowing to significantly reduce the incidence of BEA among the health personnel 19.

### Conduct in front of an AEs

In the event of AES, the accident must be declared to occupational medicine. Sometimes the accident is not reported due to an underestimation of the risk, ignorance and complexity of the procedure, lack of time, and fear of stigmatization 15, 27, 28.

The immediate course of action, in the event of a bite, is to wash the skin wound with soap and water, before dipping the finger in an antiseptic solution (Dakin) for at least 5 minutes. Similarly, in the event of blood or biological liquid splashing, the mucosa must be washed with water or physiological serum for at least 5 minutes. The goal is to eliminate as many viruses as possible inoculated at the front door.

Post-exposure treatment should preferably be initiated within 4 hours of the accident, otherwise within 48 hours at most 19. According to a systematic review, early administration of antiretroviral treatment was significantly associated with a reduced risk of HIV 29. This course of action was ignored by 68.5% of the staff questioned.

Concerning hepatitis B, the injection of serum based on specific immunoglobulin before 48 hours, the anti-HBV vaccination as well as the taking of certain antivirals can be started in unvaccinated people, or in non-responders to vaccination (assay of anti-HBs antibodies < 10 IU/L), in particular, if the source patient belongs to high-risk groups 2, 19.

For viral hepatitis C, no post-exposure treatment is recommended. In addition, monitoring of the victim must be ensured in order to begin specific treatment in the event of seroconversion or illness 19.

## Conclusion

In our study, almost half of the personnel surveyed (48.5%) were exposed, at least once, to an AES, which testifies to the extent of the problem and the need to put in place a risk management policy. infectious.

This policy must be carried out at two levels; the first concerns a priori risk management (before the occurrence of BEA), based on anti-hepatitis B vaccination, staff training in good practices related to the organization of care as well as the management of the DASRI.

The second level concerns the management of the infectious risk a posteriori that is to say after the BEA. In this context, the protocol relating to the conduct to be followed in front of an AES should be displayed in all hospital departments and known to all staff, in particular those identified as being at risk.

The establishment of an active surveillance system for BEAs is of undeniable interest. This system will not only make it possible to assess the real extent of this problem but above all to target the appropriate control measures adapted to our context.

## Figures and Tables

**Figure 1:**
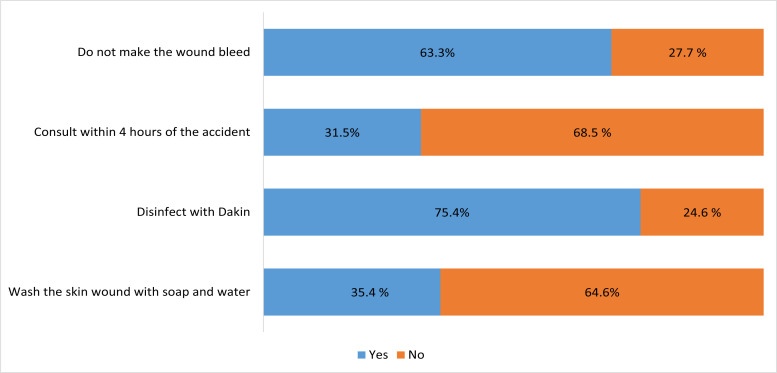
Conduct in front of an AES

## References

[1] Nouetchognou JS (2016). Accidental exposures to blood and body fluids among health care workers in a Referral Hospital of Cameroon. BMC research notes.

[2] Karé M (2018). Accidents d'exposition au sang et aux liquides biologiques. Accidents d'exposition au risque viral. EMC - Médecine d'urgence.

[3] Seeff L (1978). Type B hepatitis after needle-stick exposure: prevention with hepatitis B immune globulin: final report of the Veterans Administration Cooperative Study. Annals of Internal Medicine.

[4] Yazdanpanah Y (2005). Risk factors for hepatitis C virus transmission to health care workers after occupational exposure: a European case-control study. Clinical Infectious Diseases.

[5] Prüss-Üstün A, Rapiti E, Hutin YJ (2003). Sharps injuries: global burden of disease from sharps injuries to health-care workers.

[6] Beghdadli B (2009). Le personnel à risque d'accidents d'exposition au sang dans un-CHU de l'Ouest algérien. Sante Publique.

[7] Beghdadli B (2012). Les accidents d'exposition au sang dans un centre hospitalo-universitaire en Algérie: résultats de cinq années de surveillance (2005-2009). Archives des Maladies Professionnelles et de l'Environnement.

[8] Beghdadli B (2011). Les accidents d'exposition au sang (AES) chez les dentistes de l'ouest algérien. Journal International de la Santé au Travail.

[9] Laraqui O (2008). Évaluation des connaissances, attitudes et pratiques sur les accidents d'exposition au sang en milieu de soins au Maroc. Médecine et Maladies Infectieuses.

[10] Jahic R (2018). Epidemiological characteristics of the accidental exposures to blood-borne pathogens among workers in the hospital. Medical Archives.

[11] Berahou H (2017). Les accidents d'exposition au sang chez les étudiants en médecine de Casablanca (Maroc/span>): Analyse des connaissances et pratiques. Sante Publique.

[12] Bouhlel M (2018). Les accidents d'exposition au sang chez les jeunes médecins: fréquence et facteurs de risque. Archives des Maladies Professionnelles et de l'Environnement.

[13] Efstathiou G (2011). Factors influencing nurses' compliance with Standard Precautions in order to avoid occupational exposure to microorganisms: A focus group study. BMC nursing.

[14] Gooch BF (1995). Percutaneous exposures to HIV-infected blood: Among dental workers enrolled in the CDC Needlestick Study. The Journal of the American Dental Association.

[15] Mejdoub Y (2016). INF-11 - Fréquence et caractéristiques des accidents d'exposition au sang chez les infirmiers. Médecine et Maladies Infectieuses.

[16] Bambenongama NM, Likwela JL (2013). Connaissances, attitudes et pratiques des professionnels de santé face aux précautions standards en milieu hospitalier. Sante Publique.

[17] Tarantola A, Abiteboul D, Rachline A (2006). Infection risks following accidental exposure to blood or body fluids in health care workers: a review of pathogens transmitted in published cases. American journal of infection control.

[18] Organization, W.H. (2002). The world health report 2002: reducing risks, promoting healthy life.

[19] De Laroche M (2019). Exposition à risque de transmission virale (AES). La Revue de Médecine Interne.

[20] West DJ (1984). The risk of hepatitis B infection among health professionals in the United States: a review. The American journal of the medical sciences.

[21] Hadler S (1990). Hepatitis B virus infection and health care workers. Vaccine.

[22] Abiteboul D (2011). Vaccination des professionnels de santé: obligations et recommandations. Journal des Anti-infectieux.

[23] Denis F, Abitbol V, Aufrère A (2004). Évolution des stratégies vaccinales et couverture vaccinale contre l'hépatite B en France, pays de faible endémie. Médecine et Maladies Infectieuses.

[24] Mahoney FJ (1997). Progress toward the elimination of hepatitis B virus transmission among health care workers in the United States. Archives of Internal Medicine.

[25] Bayeux-Dunglas M, Ferreira M (2016). Vaccinations en milieu de travail. EMC-Pathologie professionnelle et de l'environnement.

[26] SF2H (2017). Actualisation des précautions standard. Établissement de santé. Établissements médicosociaux.

[27] Sellami I (2018). Les causes de la sous-déclaration des accidents d'expositions au sang. Archives des Maladies Professionnelles et de l'Environnement.

[28] Adarmouch L (2011). Fréquence des accidents exposant au sang chez les étudiants en médecine à Marrakech. Médecine et Maladies Infectieuses.

[29] Young T (2007). Antiretroviral post-exposure prophylaxis (PEP) for occupational HIVexposure. Cochrane Database of Systematic Reviews.

